# The Sick-Room

**Published:** 1884-08

**Authors:** 


### THE SICK-ROOM.

HOW to manage a sick-room so that it shall be as wholesome, as comfortable and as cheerful as the possibility of the case admits, is a study which will probably never receive the attention it merits until the "art of nursing" shall be fully recognized as forming a legitimate and important branch of the art of healing.

No error of management in a sick-room is so common as a neglect of proper temperature and ventilation; yet upon these very subjects physiologists have written more pages, and physicians spent more breath, than upon all kindred topics put together. Of course no inflexible rule can be formulated upon these points. An open window may be salvation to a man in the burning stage of a fever; at the same time it is fatal to one in the sweating stage. With all due regard for the thermometer, good sense and enlightened and careful observation should regulate these essential conditions.

Every one admits the necessity of keeping the air of a sick-room as pure as possible, yet how seldom, except in cases of contagion, do we see disinfectants about a sick-bed. Chloride of lime and carbolic acid are cheap and effective, and something of the kind should always be used wherever fresh air cannot be freely admitted.

There should always be a towel-rack in the room, or just outside, where moistened cloths can be quickly dried. Cloths wet in pure water even give out an unpleasant odor if left lying in a heap to mold or dry as they best can. Keep one stand or table for food or drinks and another for medicines. A clean newspaper often renewed makes a better covering for such tables than a cloth. Have a basin of water always at hand, that whenever a spoon or wineglass is used, it can be rinsed immediately and ready for use. Guard against irritating noise, creaking doors, chairs or shoes, also against too much light and cross lights. Holes in window-shades and gaps in shutters are often first-class nuisances. Many an invalid is tortured by a single point of piercing light, which seems, like a faithful eye, to pursue him everywhere, and to which in spite of himself he is ever returning.

A little daily change in the arrangement of furniture, a little planning for such pleasant surprises as an invalid can bear, in the way of food, company and amusement, will often be more effectual in hastening the convalesence of a patient than all the tonics that have been used in the building up of infirm humanity since the days of Esculapius.

A soft tread, a mild, persuasive word, a magnetic hand, a tact—or shall we say a genius?—for detecting the interior aspects of disease, are invaluable adjuncts of good nursing. Clatter of firearms, rattle of dishes and newspapers, loud talking and whispering, are about equally abominable.

Growing plants are usually interdicted in a sick-room, but cut flowers are great brighteners of its somberness.—[*Tribune and Farmer.*

